# The genetic architecture of type 1 diabetes over time: how well can we rely on past data to predict the future?

**DOI:** 10.1007/s00125-025-06422-6

**Published:** 2025-04-12

**Authors:** Jose Zapardiel-Gonzalo, Anette-Gabriele Ziegler, Ezio Bonifacio

**Affiliations:** 1 Institute of Diabetes Research, Helmholtz Munich, German Research Center for Environmental Health, Munich, Germany; 2 Center for Regenerative Therapies Dresden, Faculty of Medicine, TU Dresden, Dresden, Germany

**Keywords:** Genetic susceptibility, Polygenic risk scores, Type 1 diabetes

*To the Editor:* Much of our understanding of how to predict future diseases is derived from data collected years and decades ago. However, the incidence of many diseases has evolved over time. For instance, there has been a substantial increase in the incidence of type 1 diabetes, accompanied by a shift in the age distribution, with the magnitude of these changes varying across different regions globally [1]. Since the genetic composition of populations remains relatively stable over time, these shifts in disease incidence are largely attributed to environmental factors. A recent example is the COVID-19 pandemic and its associated lockdowns, which were linked to a transient increase in the incidence of type 1 diabetes [2].

The precise mechanisms by which environmental factors interact with genetic susceptibility remain unclear, as does the impact of changing disease incidence on the genetic architecture of diseases. Genetic susceptibility for type 1 diabetes is conferred by HLA class II genes and multiple genes linked to immune function, islet cell function and responses to environmental exposures such as viruses [3]. One possibility is that environment-related increases in disease incidence may elevate disease penetrance uniformly for the susceptible alleles across all risk genes. Alternatively, specific environmental factors may interact with particular risk genes and alleles, including previously unreported genes, or preferentially increase the penetrance of non-susceptible alleles, thereby altering the genetic landscape of disease over time and across regions. Indirectly supporting this, many infections have associations with genes involved in immune functions, and some of these genes also confer susceptibility to type 1 diabetes [4]. Furthermore, a previous study suggested that the genetic profile of type 1 diabetes has shifted, with higher-risk genotypes of HLA-DR and HLA-DQ, which contribute the most substantial genetic risk for the disease, becoming less prominent over time [5].

Genetic insights in type 1 diabetes have led to the development of polygenic risk scores (PRS) as a composite score for risk at multiple genetic risk regions to distinguish individuals with and without type 1 diabetes [6]. The most advanced PRS, the GRS2, could identify 77% of individuals from the Type 1 Diabetes Genetic Consortium (T1DGC) cohort who developed type 1 diabetes during childhood or adolescence (77% sensitivity) at a threshold score representing the 90th centile of the background population (90% specificity) [6]. The majority of individuals in the T1DGC cohort were of European descent and diagnosed prior to the year 2000. Given that the incidence of type 1 diabetes has increased by 50% or more over the past decades, these earlier estimates of sensitivity using PRS may no longer be accurate.

We applied the GRS2 to two cohorts of individuals of European descent who were diagnosed with type 1 diabetes before age 20 years in Germany. The first cohort included 400 individuals diagnosed between 1963 and 1999 (median year of diagnosis, 1980; median age at diagnosis, 14 years) who were the parent proband of children followed in the German BABYDIAB study [7]. Autoantibody status at type 1 diabetes onset was unknown. The second cohort comprised 1068 children and adolescents diagnosed as islet autoantibody-positive type 1 diabetes between 2008 and 2018 (median year of diagnosis, 2014; median age at diagnosis, 10 years) through the Dimelli study in Bavaria, Germany [8]. For background comparison, we analysed data from 8581 children born in Bavaria in 2019 who had no first-degree family history of type 1 diabetes, who were screened through the Global Platform for the Prevention of Autoimmune Diabetes (GPPAD) [9].

The median and the 90th percentile of the GRS2 in the background population were 10.51 and 13.36, respectively (Table 1), similar to the values observed in the UK Biobank cohort (10.46 and 13.41), where the GRS2 was validated. Using the 90th percentile as a threshold, 70.8% (95% CI 66.0, 75.1) of patients from the earlier BABYDIAB parent cohort were identified, which is significantly lower than the 77% observed in the T1DGC cohort (*p*= 0.039). In comparison, 63.8% (95% CI 60.9, 66.7) of patients in the more recent Dimelli cohort had a GRS2 above this threshold (*p*<0.0001 vs T1DGC and *p*=0.016 vs BABYDIAB parent cohort).

**Table 1:** GRS2 in a Bavarian background population and in individuals with type 1 diabetes diagnosed before and after the year 2000

Variable	Background population	German T1D BABYDIAB 1963–1999	Bavarian T1D Dimelli 2008–2018	*P* BABYDIAB vs Dimelli
Number in cohort	8581	400	1068	
Median total score	10.51	14.31	13.95	0.0035
Median HLA score	7.14	10.15	9.89	0.049
Median non-HLA score	3.53	4.19	4.10	0.016
Frequency >90th centile of background (95% CI)	10% (9.4, 10.7)	70.8% (66.0, 75.1)	63.8% (60.9, 66.7)	0.016

T1D, type 1 diabetes

To further explore temporal trends, we compared GRS2 scores between individuals diagnosed with type 1 diabetes during two time periods. The median GRS2 score was 14.31 for children diagnosed prior to 2000 and 13.95 for those diagnosed from 2008 to 2018 (*p*=0.0035). GRS2 scores were inversely correlated with the age of diagnosis in the combined cohorts (*r*=−0.061; *p*=0.02). Median GRS2 scores were lower in the recent Dimelli cohort as compared with the earlier BABYDIAB parent cohort for individuals diagnosed after age 12 years (13.80 vs 14.40; *p*=0.0009), but not for individuals diagnosed prior to age 12 years (14.02 vs 14.27; *p*=0.41). GRS2 is derived from a composite score for HLA class II and HLA interactions plus a composite score derived from SNPs outside the HLA region. A decrease in both components was observed in the later-diagnosed Dimelli cohort as compared with the earlier BABYDIAB parent cohort (HLA class II component, 10.15 vs 9.89, *p*=0.049; non-HLA component, 4.19 vs 4.10, *p*=0.016). Similar findings were obtained using the GPPAD PRS [4] that includes a smaller and slightly different panel of SNPs, with lower scores observed in the Dimelli cohort diagnosed after the year 2000 (median score, 12.83) as compared with the BABYDIAB parent cohort diagnosed before the year 2000 (median score, 13.18; *p*=0.0018). Limitations include a higher median age at diagnosis and a lack of islet autoantibody data in the earlier BABYDIAB parent cohort.

These findings suggest that the genetic architecture of childhood type 1 diabetes has likely evolved over time with more penetrance among less susceptible alleles of risk genes inside and outside the HLA class II region. Although limited by a smaller size, a difference in PRS over time was observed in older individuals with type 1 diabetes (based on age grouping), but not in the younger group. Our results show that relatively small shifts in PRS can substantially affect their disease sensitivity and underscore the need for caution when making predictions based on data from disease cases diagnosed in earlier decades. They also highlight the importance of maintaining disease registries with bioresource materials to facilitate ongoing investigations into the changing epidemiology and genetic underpinnings of type 1 diabetes across different regions and racial groups.

## Data Availability

The de-identified individual data that underlie the results can be shared. Requests will be honoured from researchers who provide a methodologically sound proposal and who complete a Data Use Agreement with the Helmholtz Zentrum München. Requests should be directed by email to the corresponding author.

## Abbreviations

GPPAD: Global Platform for the Prevention of Autoimmune Diabetes
PRS: Polygenic risk scores
T1DGC: Type 1 Diabetes Genetic Consortium
